# Supplementary material for: Impact of Physical Activity Counselling on Children with Medical Conditions and Disabilities and Their Families
Source: Children (Basel). 2023 Jul 27;10(8):1293. doi: 10.3390/children10081293 (PMC10453572; doi:10.3390/children10081293)
Supplement: Supplementary file 1 [file children-10-01293-s001.zip › File S1 Child Interview Guide 2021-05-10.pdf]

Semi-Structured Interview Guide (Child):

Preamble Script:
Thank you for being in our study about the physical activity counselling at CHEO. Being active is important for children's health, but many children and youth at CHEO find it difficult to be active. We offer physical activity counselling to help children and youth be more confident and have fun during physical activity. The aim of this study is to understand what children and parents think about the physical activity counselling that CHEO provides.

Before beginning the interview, it is important that you know that your participation in this research is a choice. You can choose to participate, you can choose not to participate and you can choose to stop at any time, even after the interview has started. In addition, you should know that the information you provide during the interview is confidential for the research study. That means we will keep your information private and will not share what you tell us with anyone, including your clinical care team, except as required by law.

1. What do you remember from physical activity counselling?
   Prompts:
   a. Why do you think you remember that the best?
   b. Do you still use anything that you learned through physical activity counselling?
   c. How often do you think about or apply the things you learned in physical activity counselling?

2. How did the counselling change your physical activity?
   Prompts:
   a. How active are you today?
   b. Is your activity level today impacted by your participation in physical activity counselling?
   c. What did physical activity counselling not impact?
   d. Do you think that physical activity counselling made a difference in your life today? Why or why not?

3. What was helpful?
   Prompts:
   a. What were your main takeaways from participating in physical activity counselling?
   b. What did you enjoy about the experience?
   c. Did you feel supported throughout the process? Why or why not?
   d. Is there anything you really enjoyed?

4. What could be improved?
   Prompts:
   a. What do you think could be added to support you better?
   b. What was not helpful?

    c. Is there anything you really did not enjoy?
    d. Would you change the format?
    e. What do you think would have made a bigger impact?

5. Would you do it again?
   Prompts:
    a. Why would you like to do it again?
    b. Would you do anything differently if you were to do it a second time?

6. Would you recommend that other children or families do it?
   Prompts:
    a. Why do you think other children should do it?
    b. Should we do anything differently if other children were to do it?
