# Supplementary material for: Impact of Physical Activity Counselling on Children with Medical Conditions and Disabilities and Their Families
Source: Children (Basel). 2023 Jul 27;10(8):1293. doi: 10.3390/children10081293 (PMC10453572; doi:10.3390/children10081293)
Supplement: Supplementary file 1 [file children-10-01293-s001.zip › File S2 Parent Interview Guide 2021-05-10.pdf]

Semi-Structured Interview Guide (Parent):

Preamble Script:
Thank you for being in our study about the physical activity counselling at CHEO. Being active is important for children's health, but many children and youth at CHEO find it difficult to be active. We offer physical activity counselling to help children and youth be more confident and have fun during physical activity. The aim of this study is to understand what children and parents think about the physical activity counselling that CHEO provides.

Before beginning the interview, it is important that you know that your participation in this research is a choice. You can choose to participate, you can choose not to participate and you can choose to stop at any time, even after the interview has started. In addition, you should know that the information you provide during the interview is confidential for the research study. That means we will keep your information private and will not share what you tell us with anyone, including your clinical care team, except as required by law.

1. What do you remember from your child's physical activity counselling?
   Prompts:
   a. Why do you think you remember that the best?
   b. Does your child still use anything that they learned through physical activity counselling?
   c. How often do you think they think about or apply the things they learned in physical activity counselling?

2. How did the counselling change your child's physical activity?
   Prompts:
   a. How active is your child today?
   b. Is your child's activity level today impacted by their participation in physical activity counselling?
   c. What did physical activity counselling not impact?
   d. Do you think that physical activity counselling made a difference in your child's life today? Why or why not?

3. How did the counselling change your understanding of your child's physical activity?
   a. Are you more confident about the activities appropriate for your child?
   b. Do you think you are better able to support and encourage your child's physical activity?

4. What was helpful?
   Prompts:
   a. What do you think your child took away from the experience?
   b. What were your main takeaways from participating in physical activity counselling?
   c. What do you think your child enjoyed about the experience?

d. What did you enjoy about the experience as a parent?
e. Do you think your child felt supported throughout the process? Why or why not?
f. Did you feel supported throughout the process? Why or why not?
g. Is there anything your child really enjoyed?
h. Is there anything you really enjoyed?

5. What could be improved?
   Prompts:
   a. What do you think could be added to support your child better?
   b. What do you think could be added to support you better?
   c. What was not helpful for your child?
   d. What was not helpful for you?
   e. Is there anything your child really did not enjoy?
   f. Is there anything you really did not enjoy?
   g. Would you change the format?
   h. What do you think would have made a bigger impact for your child?
   i. What do you think would have made a bigger impact for yourself?

6. Would you do it again?
   Prompts:
   a. Why would you like to do it again?
   b. Would you do anything differently if you were to do it a second time?

7. Would you recommend that other children or families do it?
   Prompts:
   a. Why do you think other children/families should do it?
   b. Should we do anything differently if other children/families were to do it?
